# Detecting Coronary Artery Disease Using Rest Seismocardiography and Gyrocardiography

**DOI:** 10.3389/fphys.2021.758727

**Published:** 2021-12-02

**Authors:** Parastoo Dehkordi, Erwin P. Bauer, Kouhyar Tavakolian, Zhen G. Xiao, Andrew P. Blaber, Farzad Khosrow-Khavar

**Affiliations:** ^1^Heart Force Medical Inc., Vancouver, BC, Canada; ^2^School of Electrical Engineering and Computer Science, University of North Dakota, Grand Forks, ND, United States; ^3^Biomedical Physiology and Kinesiology Department, Simon Fraser University, Vancouver, BC, Canada

**Keywords:** seismocardiography, gyrocardiography, coronary artery disease (CAD), cardiac mechanical activity, angiography

## Abstract

In this study, we present a non-invasive solution to identify patients with coronary artery disease (CAD) defined as ⩾50% stenosis in at least one coronary artery. The solution is based on the analysis of linear acceleration (seismocardiogram, SCG) and angular velocity (gyrocardiogram, GCG) of the heart recorded in the x, y, and z directional axes from an accelerometer/gyroscope sensor mounted on the sternum. The database was collected from 310 individuals through a multicenter study. The time-frequency features extracted from each SCG and GCG data channel were fed to a one-dimensional Convolutional Neural Network (1D CNN) to train six separate classifiers. The results from different classifiers were later fused to estimate the CAD risk for each participant. The predicted CAD risk was validated against related results from angiography. The SCG z and SCG y classifiers showed better performance relative to the other models (*p* < 0.05) with the area under the curve (AUC) of 91%. The sensitivity range for CAD detection was 92–94% for the SCG models and 73–87% for the GCG models. Based on our findings, the SCG models achieved better performance in predicting the CAD risk compared to the GCG models; the model based on the combination of all SCG and GCG classifiers did not achieve higher performance relative to the other models. Moreover, these findings showed that the performance of the proposed 3-axial SCG/GCG solution based on recordings obtained during rest was comparable, or better than stress ECG. These data may indicate that 3-axial SCG/GCG could be used as a portable at-home CAD screening tool.

## 1. Introduction

Heart disease is the number one leading cause of death worldwide, with coronary artery disease (CAD) accounting for about 44% of these deaths (GBD 2017 Disease and Injury Incidence and Prevalence Collaborators, 2018). CAD defines a family of diseases caused by build-up plaque in coronary arteries, blood vessels running over the surface of the heart to supply oxygenated blood to the myocardium. The plaque, made up of fat, cholesterol, calcium, and other substances in the blood, gradually hardens and narrows the arteries. Plaque build-up may cause permanent artery occlusion leading to acute myocardial infarction. As a result, it is of extreme importance to diagnose CAD in its early stages, before myocardial infarction occurs.

In most cases, assessment and diagnosis of CAD start after the patient experiences symptoms such as chest pain, shortness of breath, and fatigue. In such cases, the patients are first referred for rest and stress electrocardiography (ECG) test. Subsequently, further tests such as coronary computed tomography angiography (CCTA) or coronary angiography are used to localize where the occlusion has occurred (Ashley and Niebauer, 2004).

While rest and stress ECG tests are extensively used for the diagnosis of CAD, in many cases, diagnosis using only the morphological changes of ECG is not straightforward, especially when the disease is in an early stage of development. On a rest ECG, the presence of ST-segment elevations suggests that the patient is most likely suffering from ST-elevation myocardial infarction. However, some patients are suffering from myocardial infarction who do not exhibit ST elevations on an ECG. During an ECG stress test, a 12-lead ECG is recorded to assess the heart's ability to respond to external stress induced by exercise or intravenous pharmacological stimulation. When the heart is under stress, developing plaque may induce changes in the ECG, mostly as either depression or an elevation of the ST segment. An exercise ECG is non-invasive, inexpensive, and widely available and despite its low sensitivity of 60%-70% (Al-Shehri et al., 2011; Benjamin et al., 2018), it is still the primary choice for most cases of suspected CAD.

Cardiac imaging such as coronary angiography or CCTA offers greater sensitivity and specificity than an exercise test. Cardiac imaging is currently the gold standard for the diagnosis of CAD, but it can be expensive and not widely available. In the case of angiography, it is invasive, introduces dye into the body, and may involve harmful radiation (Al-Shehri et al., 2011).

A monitoring device that non-invasively and accurately screens cardiac function could improve early detection of coronary artery stenosis before it develops into ischemia or myocardial infarct. This would lead to improved health status among individuals and, subsequently, fewer medical complications. In this study, we examined a methodology for detecting CAD with a sensor designed to measure chest vibrations induced by mechanical activity of the heart during rest.

The heart, in each cardiac cycle, twists forward and taps the chest wall. This induces chest vibrations which can be measured with a joint accelerometer-gyroscope mounted on the sternum. The accelerometer non-invasively captures the linear acceleration, or seismocardiogram (SCG), of the chest while the gyroscope records the angular velocity or gyrocardiogram (GCG).

An SCG is commonly recorded dorsoventrally using an accelerometer placed on the sternum close to the xiphoid process. SCG was initially recommended, in the early 1960s, for monitoring heart rate variability (Baevskii et al., 1964). In the late 80s and early 90s, SCG was used as a technology for measuring the myocardium motion during ventricular contraction, and during early and late ventricular filling (Salerno and Zanetti, 1990, 1991). A study conducted by Crow et al. suggested that the fiducial points of the dorsoventral SCG were associated with aortic and mitral valve opening and closure events (Crow et al., 1994) which were further investigated in a recent study (Dehkordi et al., 2019b). In studies conducted in 1990 and 1991, SCG was suggested as a non-invasive technology for detecting coronary artery disease (Salerno et al., 1990, 1991). In a study done by Salerno et al., the seismographic changes associated with coronary artery stenosis were investigated in 35 patients during coronary angioplasty (Salerno and Zanetti, 1991). The findings were consistent with the hypothesis that the SCG changes were due to ischemic changes in ventricular wall motion.

Several studies were later conducted to assess the ability of exercise SCG for detecting CAD. Salerno et al. studied the morphology of exercise SCG in patients with ⩾50% coronary artery stenosis (Salerno et al., 1992). Changes in the morphology of SCG before and immediately after exercise were reported as being significant during isovolumetric contraction up to the occurrence of aortic valve opening. Their findings suggested that exercise SCG in conjunction with 12-channel ECG improved the sensitivity of detection of coronary artery stenosis compared to ECG alone. These findings were later confirmed by the work of Korzeniowska-Kubacka et al. (2005).

More recently, GCG has been introduced as a non-invasive method for capturing the angular velocity of the chest induced by heart rotation using a gyroscope placed on the sternum. The angular velocity of the chest can be described as the rate of angular displacement of the chest in terms of the speed of rotation and the axis about which it is rotating. A uni-axial gyroscope measures the angular velocity acting along a single measuring axis while a 3-axial gyroscope, formed by three orthogonal uni-axial gyroscopes, measures the angular velocity along the x-axis, y-axis, and z-axis. GCG has been investigated as a new technology for indicating the valvular opening and closing points and consecutively for measuring the cardiac timing intervals (Jafari Tadi et al., 2016, 2017; Dehkordi et al., 2020).

In a recent study (Dehkordi et al., 2019a), we developed a machine-learning algorithm to automatically detect CAD using the same database as the one (Salerno et al., 1992). The database consisted of the SCG signals recorded from 185 individuals at rest and immediately after exercise. We implemented two separate models for identifying individuals with CAD using the rest and exercise SCG. The models were validated against related results from angiography. For the rest model, accuracy was 74%, and sensitivity and specificity were estimated as 75 and 72%, respectively. For the exercise model, accuracy, sensitivity, and specificity were 81, 82, and 84%, respectively. Both rest and exercise models were able to detect CAD with comparable accuracy, sensitivity, and specificity. Performance of exercise SCG was found to be better when compared to stress-ECG, which is identical to stress-echocardiography and CCTA. However, similar to the exercise ECG, the exercise SCG would be restricted to medical facilities; therefore, in the current study, we are investigating the ability of the rest SCG/GCG to detect CAD.

The purpose of the current investigation was to assess whether the analysis of combined SCG and GCG recordings can improve the detection of CAD without the need to stress the heart. We designed and conducted a multicenter study and collected a new database consisting of 3-axial SCG and GCG signals from individuals with and without CAD. We implemented six separate classifiers using features extracted from 6-channel data of SCG and GCG. The results from different classifiers were later fused to predict the final CAD risk for each patient. The predicted CAD risk was then validated against related results from angiography.

## 2. Materials and Methods

### 2.1. Data Set

#### 2.1.1. Participants

In total 326 individuals were recruited for this study. The dataset was collected during a multi-center study conducted in Zurich, Switzerland, and in Vancouver, Canada.

In Zurich, 157 patients (age: 63.71 ± 11.5, 30 females) were recruited for this study. The patients were suspected of having CAD and were referred to the University Hospital of Zurich for catheter coronary angiography. The occlusion of more than 50% in at least one coronary artery was considered as CAD. Among the 157, 7 participants (4.4%) were excluded from the study due to lack of information from angiography, very poor quality ECG, SCG, or GCG signals, or unwillingness to continue the study. On the remaining 150 participants, 126 patients were diagnosed with significant CAD by coronary angiography while in 24 out of 150 patients no significant CAD was reported. The protocol of this study was approved by the University Hospital of Zurich; the study was carried out following the Swiss legal requirements and procedures involving human participants with written informed consent from all subjects.

In Vancouver, 169 healthy, male and female adults between 19 and 85 years of age (age: 42.7 ± 15.7, 85 females) were recruited for this study. Participants with a known history of cardiovascular, respiratory, or major musculoskeletal injuries were excluded from recording. Participants were later scanned with echocardiography to detect any visible cardiac anomalies including valvular regurgitations, pre-existing congenital heart disease, and abnormal motion of the myocardium. Among the 169, 11 participants (6.5%) were excluded from the study due to very poor quality ECG, SCG or GCG signals, unwillingness to continue the study or abnormal motion of myocardium (1 person). The remaining 158 were labeled as non-CAD. This study was carried out following the recommendations of Simon Fraser University policies and procedures involving human participants with written informed consent from all subjects. The protocol was approved by the Office of Research Ethics at Simon Fraser University, Vancouver, Canada.

#### 2.1.2. Data Acquisition

**Figure 1:**
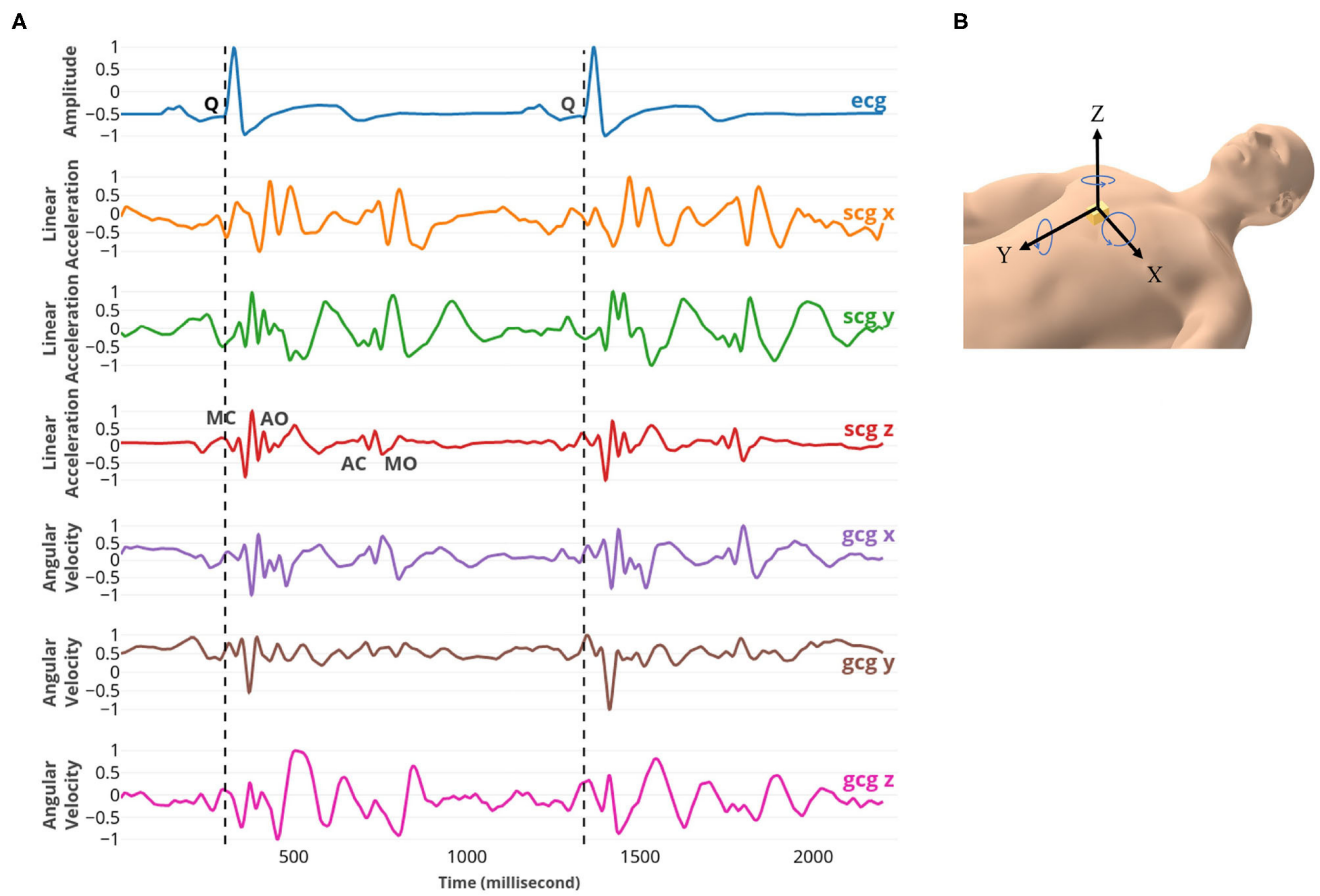
**(A)** From top to bottom: ECG, SCG x, y, and z, and GCG x, y, and z signals captured simultaneously. The fiducial points MC, AO, AC, and MO were marked on SCG z. MC and MO points correspond to mitral valve closure and opening; AC and AO points correspond to the aortic valve closure and opening. **(B)** The position and the direction of the 3-axial micro-electro-mechanical (MEMS) joint accelerometer-gyroscope sensor.

To measure the heart's mechanical motion, a low-noise 3-axial micro-electro-mechanical (MEMS) joint accelerometer-gyroscope sensor (ASC GmbH, ASC IMU 7.002LN.0750, Germany) was used to record SCG and GCG. The dynamic range of the accelerometer and gyroscope were set ± 2 g and ±75 °*s*^−1^ with an RMS noise of 7μ*gHz*^−1/2^ and 0.02 °*s*^−1^*Hz*^−1/2^, respectively. The sensor was mounted on the sternum with the x-axis pointed laterally from left to right, the y-axis pointed from head to foot, and the z-axis pointed from back to front while participants lay supine. Simultaneously, a reference two-lead ECG (iWorx Systems, Inc., IX-BIO8-SA, NH, USA) was recorded. All recordings were conducted with the iWorx data acquisition system (iWorx Systems, Inc., IX-416, NH, USA), sampled at 1 kHz with 16-bit resolution (Figure 1).

All data recordings in Vancouver were performed at the Aerospace Physiology Lab at Simon Fraser University, Canada. In Switzerland, data collection was conducted in the Institute of Diagnostic and Interventional Radiology of the University Hospital Zurich.

### 2.2. Data Processing

**Figure 2:**
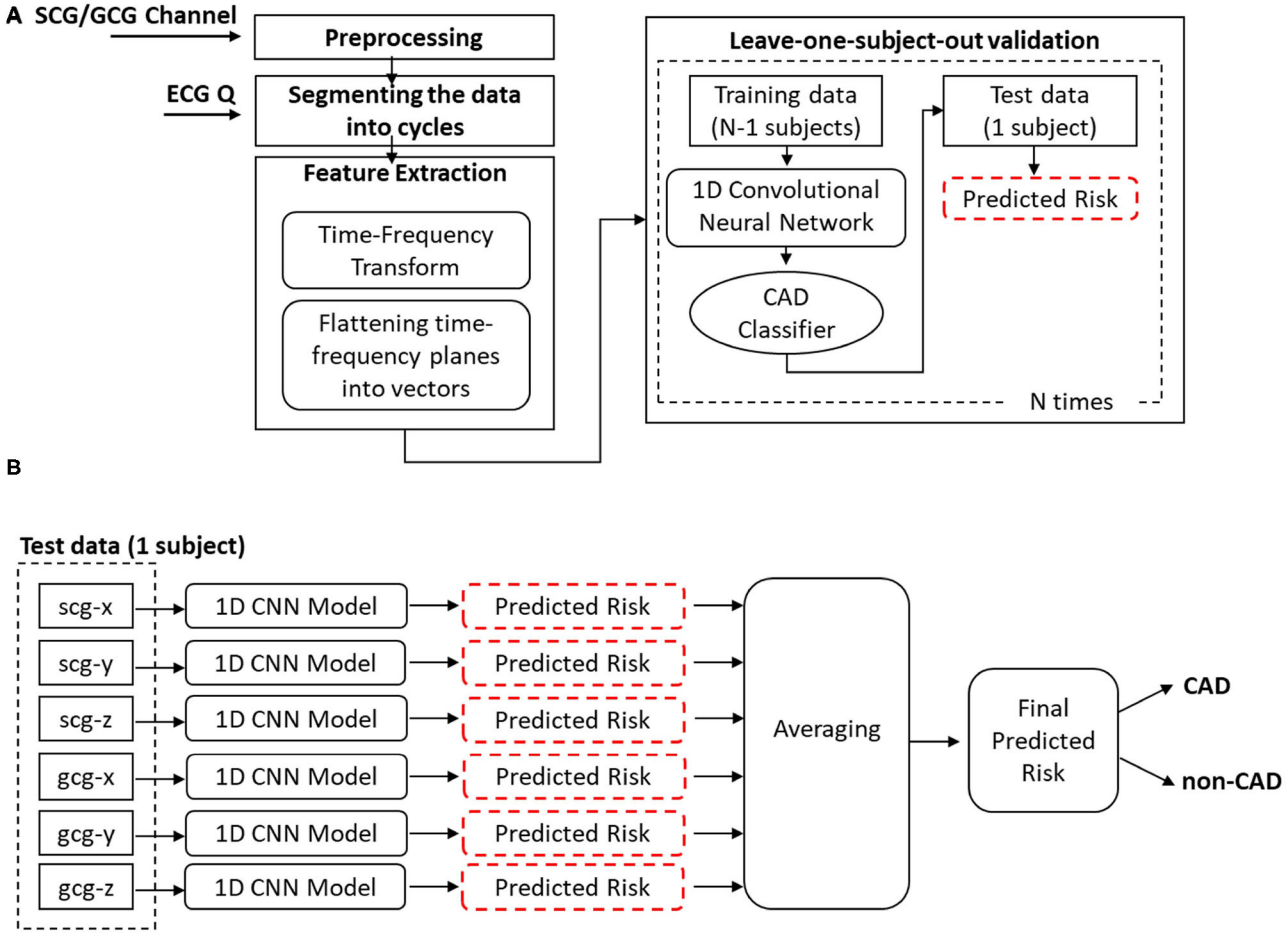
**(A)** Different steps of methodology **(B)** Ensemble learning.

Data analysis was performed in three main steps: 1) feature extraction, 2) model training, and 3) ensemble learning and validation (Figure 2).

In the first step, after preprocessing, each SCG and GCG channel of data was segmented into the cardiac cycles using the ECG Q. For each cycle, a two-dimensional matrix of time-frequency features was extracted using the synchrosqueezing transform and was later flattened to a one-dimensional grayscale vector. In the model training step, the sets of grayscale vectors extracted from six SCG/GCG channels were separately fed to a one-dimensional Convolutional Neural Network (1D CNN) to get six different classifiers [three classifiers for SCG channels (CAD_*scg*_*x*_, CAD_*scg*_*y*_, and CAD_*scg*_*z*_) and three classifiers for GCG channels (CAD_*gcg*_*x*_, CAD_*gcg*_*y*_, and CAD_*gcg*_*z*_)]. In the last step, the results from different classifiers were fused to estimate the final CAD risk for each patient. The leave-one-subject-out cross-validation was employed to validate the solution.

#### 2.2.1. Preprocessing

A zero-phase high-pass Butterworth filter with an order of 5 and the cut-off frequency of 0.5 Hz was applied to the SCG and GCG signals to remove baseline wander. Subsequently, the average was removed, and the signals were normalized between –1 and 1. All signals were downsampled to 250 Hz using a Chebyshev Type I filter with the order of 8.

#### 2.2.2. Feature Extraction

For extracting features from SCG/GCG signals, the following steps were taken (Figure 2A):

1. The onset of left ventricular depolarization (the onset of QRS complex on ECG, and in particular the ECG Q) were detected using the Pan-Tompkin algorithm (Pan and Tompkins, 1985). The ECG Q points were used to segment the SCG and GCG signals into cardiac cycles.
2. Due to heart rate variability, the cardiac cycles had different lengths and consequently different numbers of samples. All cycles were linearly interpolated, taking place at 250 equally spaced points to have the cycle with the same size.
3. After interpolation, 10 consecutive interpolated cycles were concatenated to get a time series of the length of 2,500 samples.
4. A time-frequency transform called synchrosqueezing transform (SST) (Daubechies et al., 2011) was used to determine the frequency components of each time series at each time sample. SST is a combination of wavelet analysis and a reallocation method that sharpens the time-frequency representation by allocating its points to other locations in the time-frequency plane (Thakur et al., 2013). Applying the SST resulted in a plane, T, of the size of 178 by 2,500.
5. T was divided into 10 matrices of size 178 by 250. As such, each matrix represented the time-frequency components of one cycle. Later the information related to the frequencies greater than 40 Hz were discarded, resulting in the matrices of size 150 by 250.
6. Each matrix was averaged in time and frequency dimensions using the moving windows of 5 and 10 samples, respectively, which reduced the size of matrices to 30 by 25. The length of the moving widows has been tuned in the validation data set using a grid search strategy. The matrices were converted to the intensity images that contained values in the range 0 (black) to 1 (white); the images were later flattened into vectors of size 750. These vectors were used to train and validate the one-dimensional Convolutional Neural Network (1D CNN) classifiers.

#### 2.2.3. Deep Learning Approach

##### 2.2.3.1. 1D CNN Architecture

**Figure 3:**
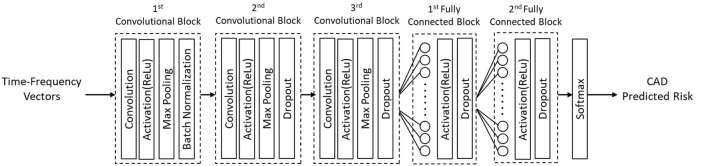
1D CNN architecture proposed for training the classifiers.

An architecture based on the one-dimensional Convolutional Neural Network (1D CNN) was proposed for training the classifiers (Figure 3). The standard CNN, also called 2D CNN, was initially introduced as a deep learning architecture for analyzing image data. The CNN extracts the spatial features of the data by using sliding kernels. In 2D CNN, the kernels slide in two dimensions while the kernels in 1D CNN slide in one dimension. It makes the 1D CNN a powerful tool for analyzing time-series data which has spatial characteristics only in one dimension.

The proposed model contained three convolutional blocks, two fully connected layers, and a Softmax layer as the output prediction layer. The convolutional block consisted of a convolutional layer, an activation layer with Rectified Linear Unit (ReLU) function, and a max-pooling layer. A batch normalization layer was added to the first convolutional block after ReLU activation to normalize the input layer. A dropout layer was applied to the second and third conventional blocks (Figure 3).

The first conventional layer had 32 filters, and the second and third conventional layers each had 16 filters. The kernel size in all layers was set to 10. The pooling layers with the kernel sizes of two were added to downsample the convolutional output. The output of conventional blocks was flattened to one long vector and passed through two fully connected layers. Each fully connected layer contained 1,000 nodes, a ReLU activation function, and a dropout layer of rate 0.5. The Softmax function in the last layer was used to estimate the prediction probability over the two classes of CAD and non-CAD.

##### 2.2.3.2. Training

The leave-one-subject-out method was employed to train the models and evaluate their performance. First, the dataset was split into Train (features of N-1 participants) and Test (features of 1 participant). After that, we kept aside the Test set and randomly assigned 80% of the Train dataset as the actual Train set and the remaining 20% as the Validation set. The 1D CNN was then iteratively trained and validated on these different sets N times. The training was carried on using the adaptive moment estimation (Adam) optimizer and the binary cross-entropy as the loss function. The performance of models was evaluated on the Validation set for 100 epochs, and the best model with the highest accuracy on the Validation set was chosen. This model later was applied to the data of the Test dataset to predict the probability of belonging to the CAD class. This probability is called predicted CAD risk.

##### 2.2.3.3. Ensemble Learning

The predictions from different classifiers were combined to estimate the final CAD risk for each patient (Figure 2B). The classifiers CAD_*all*_*axes*_, CAD_*scg*_*axes*_ and CAD_*gcg*_*axes*_ were formed from averaging the predicted risk estimated from all six classifiers, three SCG classifiers (CAD_*scg*_*x*_, CAD_*scg*_*y*_, CAD_*scg*_*z*)_ and three GCG classifiers (CAD_*gcg*_*x*_, CAD_*gcg*_*y*_, CAD_*gcg*_*z*)_, respectively.

##### 2.2.3.4. Performance Evaluation

The performance of the classifiers was evaluated in terms of area under the receiver operating characteristic curve (AUC), F1-score, sensitivity, specificity, positive predictive value (PPV), and negative predictive value (NPV). In addition, for each model, the discrimination slope was estimated as the difference in the average predicted probabilities between two classes of CAD and non-CAD.

## 3. Results

The AUCs for all models were estimated to measure the overall performance of the classifiers (Table 1). The individuals with an overall predicted risk greater than 0.5 were classified as CAD. Accordingly, the F1-score, sensitivity, specificity, positive predictive value (PPV), and negative predictive value (NPV) were estimated.

**Table 1:** Overall classification performance for the 6-channel model (CAD_*all*_*axes*_), the 3-channel SCG (CAD_*scg*_*axes*_), the 3-channel GCG (CAD_*gcg*_*axes*_), three one-channel SCG (CAD_*scg*_*x*_, CAD_*scg*_*y*_, CAD_*scg*_*z*_), and three one-channel GCG (CAD_*gcg*_*x*_, CAD_*gcg*_*y*_, CAD_*gcg*_*z*_).

**Model**	**AUC (95% CI)**	**F1**_**score**	**Sensitivity (95% CI)**	**Specificity (95% CI)**	**PPV (95% CI)**	**NPV (95% CI)**	**Discrimination slope**
CAD_*all*_*axes*_	0.92 (0.89–0.96)	0.85	96% (93–99)	76% (70–83)	76% (69–82)	96% (93–98)	0.54
CAD_*scg*_*axes*_	0.93 (0.90–0.96)	0.84	98% (95–99)	74% (67–80)	74% (67–81)	97% (95–99)	0.61
CAD_*gcg*_*axes*_	0.89 (0.85–0.93)	0.84	90% (85–96)	78% (71–84)	76% (70–82)	91% (87–96)	0.46
CAD_*scg*_*x*_	0.88 (0.84–0.92)	0.81	94% (90–98)	72% (65–79)	72% (65–79)	94% (90–98)	0.56
CAD_*scg*_*y*_	0.94 (0.90–0.98)	0.86	94% (90–98)	78% (72–84)	77% (71–84)	95% (91–98)	0.65
CAD_*scg*_*z*_	0.91 (0.88–0.94)	0.85	92% (88–97)	78% (72–84)	78% (71–84)	92% (88–97)	0.65
CAD_*gcg*_*x*_	0.86 (0.82–0.91)	0.81	73% (65–81)	82% (77–89)	76% (69–84)	80% (74–86)	0.45
CAD_*gcg*_*y*_	0.83 (0.79–0.88)	0.78	81% (74–88)	73% (66–79)	70% (62–77)	83% (77–89)	0.42
CAD_*gcg*_*z*_	0.83 (0.79–0.88)	0.79	87% (81–93)	72% (65–79)	71% (63–78)	88% (82–93)	0.5

*The F1-score, accuracy, sensitivity, specificity, positive predictive value (PPV), and negative predictive value (NPV) were estimated for the threshold of 0.5*.

Considering AUC and F1-score, the CAD_*scg*_*z*_ and CAD_*scg*_*y*_ showed better performance relative to the other models (p-value < 0.05). Considering NPV, CAD_*scg*_*axes*_ demonstrated the best performance. In general, the SCG models (CAD_*scg*_*x*_, CAD_*scg*_*y*_, CAD_*scg*_*z*_, CAD_*scg*_*axes*)_ perform better in identifying CAD from non-CAD compared to the GCG models (CAD_*gcg*_*x*_, CAD_*gcg*_*y*_, CAD_*gcg*_*z*_, CAD_*gcg*_*axes*)_. The CAD_*all*_*axes*_ using the data from all channels would not achieve higher performance relative to the other models (Table 1).

**Figure 4:**
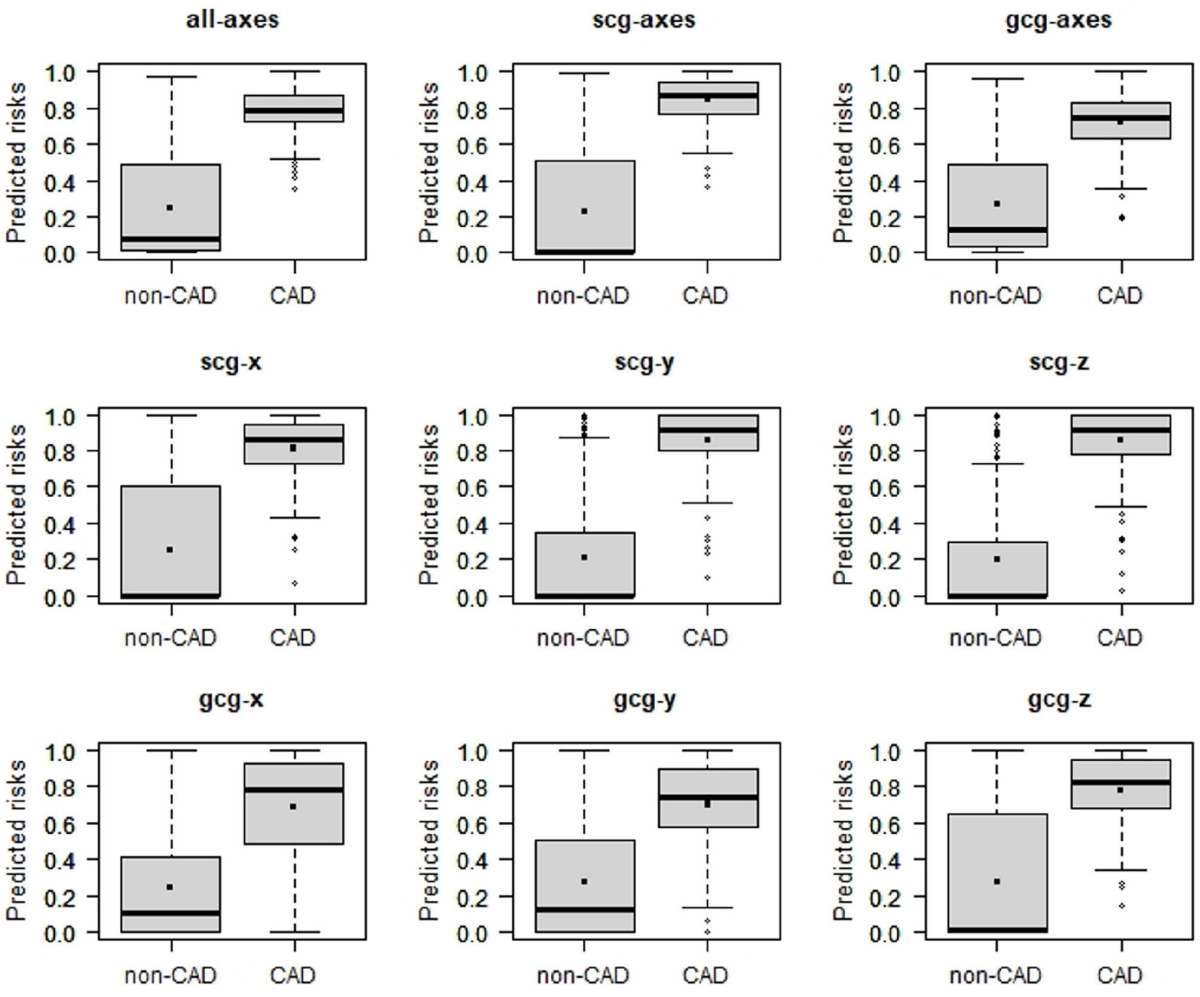
Box plots of predicted risk of individuals with and without CAD estimated 6-channel model (CAD_*all*_*axes*_), the 3-channel SCG (CAD_*scg*_*axes*_), the 3-channel GCG (CAD_*gcg*_*axes*_), three one-channel SCG (CAD_*scg*_*x*_, CAD_*scg*_*y*_, CAD_*scg*_*z*_), and three one-channel GCG (CAD_*gcg*_*x*_, CAD_*gcg*_*y*_, CAD_*gcg*_*z*)_.

Among all models, CAD_*scg*_*y*_ and CAD_*scg*_*z*_ have attained the greatest discrimination slope of 0.65 (Figure 4, Table 1).

## 4. Discussion

We have proposed a novel and non-invasive solution for identifying the patients with more than 50% occlusion in at least one coronary artery by analyzing the heart's mechanical activity. The human heart rotates along its long axis in two different directions on the base and apex, causing a very complicated twist and wringing during each cycle. The proposed solution analyzed both the linear acceleration (SCG) and angular velocity (GCG) and in three different axes (x, y, and z) to examine the effect of coronary artery disease in both the heart motion and the heart twist. SCG and GCG were recorded using a joint 3-axial accelerometer/gyroscope inertial measurement unit (IMU) sensor mounted on the chest. Six different deep learning models were developed using the SCG/GCG data channels to predict the CAD risk for each participant. In addition, CAD_*all*_*axes*_, CAD_*scg*_*axes*_, and CAD_*gcg*_*axes*_ models were developed from the fusion of all six SCG/GCG models, three SCG models, and three GCG models, respectively. The performance of the models was validated against the gold standard, angiography. The performance of the models in terms of AUC and F1-score was also compared to each other to examine the ability of each data channel of SCG/GCG or their combination in detecting CAD. The models delivered the AUC, and F1-score ranged from 0.83 to 0.92 and 0.78 to 0.86, respectively.

The finding of this study showed that the models based on the three SCG data channels (CAD_*scg*_*x*_, CAD_*scg*_*y*_, CAD_*scg*_*z*_, CAD_*scg*_*axes*_) in general perform better in identifying CAD compared to the GCG models (CAD_*gcg*_*x*_, CAD_*gcg*_*y*_, CAD_*gcg*_*z*_, CAD_*gcg*_*axes*_). It may imply that the change in vibration characteristics (time-frequency) due to coronary artery disease is more significant in the linear acceleration data than the rotational velocity recorded from the sternum. Among SCG models, CAD_*scg*_*y*_ and CAD_*scg*_*z*_ demonstrated almost the same performance with the highest values for AUC and F1-score (0.92 and 0.86, respectively). However, the model based on the three-channel SCG (CAD_*scg*_*axes*_) provided the greatest value for NPV. NPV is the probability that a person with a negative test result (non-CAD, in this study) is truly free of disease. High NPV achieved by CAD_*scg*_*axes*_ may suggest this model as the most reliable one for screening CAD, among others.

The CAD_*all*_*axes*_ model, which was an ensemble of all six models, would not achieve higher performance relative to the other models. A study conducted by Dehkordi et al. (2020) suggested that using SCG and GCG recordings together could provide the opportunity to estimate cardiac time intervals more accurately and make it possible to calculate the Tei Index as an indicator of myocardial performance. However, the results of this study may suggest that combining the analysis of GCG and SCG would not provide better performance in detecting CAD.

In our recent study (Dehkordi et al., 2019a), we implemented two separate models for identifying individuals with CAD using the rest and exercise SCG. The AUC was estimated as 0.77 and 0.91 for the rest and exercise models, respectively. For the rest model, accuracy was 74%, and sensitivity and specificity were estimated as 75 and 72%, respectively. For the exercise model, accuracy, sensitivity, and specificity were 81, 82, and 84%, respectively. In the current study, the SCG and GCG recordings were obtained during the rest, and the trained models of CAD_*all*_*axes*_, CAD_*scg*_*axes*_, CAD_*scg*_*y*_, and CAD_*scg*_*z*_ still achieved better performance with the higher AUC (ranged from 0.91 to 0.93), accuracy (ranged from 82% to 85%), and sensitivity (ranged from 92 to 98%) relative to the same metrics reported for both rest and exercise SCG in our previous study (Dehkordi et al., 2019a). However, the model based on the exercise SCG revealed higher specificity (84%) compared to those reported for CAD_*all*_*axes*_, CAD_*scg*_*axes*_, CAD_*scg*_*y*_, and CAD_*scg*_*z*_ (ranged from 72 to 78%). It suggests that the exercise SCG provided better performance in correctly identifying people without CAD. In other words, exercise SCG provided a higher true negative rate.

In our previous study, the analyses of ECG showed that for the patients with ⩾50% stenosis in at least one coronary artery, the sensitivity of exercise ECG was 70%, and for the patients without significant coronary artery stenosis, the specificity for exercise ECG was 55%; total accuracy of exercise ECG was 65%. Comparing these results shows that the CAD_*all*_*axes*_, CAD_*scg*_*axes*_, CAD_*scg*_*y*_, and CAD_*scg*_*z*_ models provided better performance in identifying patients with CAD compared to the exercise ECG.

Performance of the models we trained in this study is comparable with the performance of coronary computed tomography angiography (CCTA). Sensitivity and specificity of CCTA are stated to be between 85 and 90% and 64 and 90%, respectively. However, CCTA has a very high negative predictive value, especially in low to intermediate-risk subjects (Al-Shehri et al., 2011). Furthermore, CCTA is only available in specialized centers and it is by far more expensive compared to SCG examination.

An evidence-based analysis of more than 120 publications was recently conducted to determine the accuracy of stress echocardiography with regard to CAD. Overall pooled sensitivity of 80% (95% CI: 0.77–0.82) and specificity of 84% (95% CI: 0.82–0.87) were reported using coronary angiography as the reference standard (Medical Advisory Secretariat, 2010). The models we trained in this study showed higher sensitivity (92–98%) to those reported for the stress echocardiography. The stress echocardiography, though, revealed higher specificity (84%) compared to those reported for SCG and GCG models (ranged from 72 to 78%). However, in our opinion, recording SCG is more convenient than performing a stress echocardiography. Furthermore, analysis of SCG recordings is much easier than interpreting echocardiographic images.

The performance of the CAD models based on SCG and GCG are higher compared to those calculated for the exercise ECG. Several studies showed that the sensitivity of the exercise ECG ranged between 68 and 75% (Al-Shehri et al., 2011; McLellan and Prior, 2012) and the specificity ranged from 70 to 77% (McLellan and Prior, 2012). Besides, there is a considerable drawback that an exercise ECG test can only be performed by a trained physician. In contrast to the exercise ECG, a rest SCG can be recorded by individuals without the medical background.

The results of previous studies (Salerno et al., 1992; Dehkordi et al., 2019a) showed that an exercise SCG provided better performance in identifying patients with CAD compared to the exercise ECG. However, similar to an exercise ECG, an exercise SCG would be restricted to medical facilities under the supervision of a trained physician (e.g., a cardiologist) due to the risk of stress-induced cardiac events. The findings of the current study show that the performance of the proposed solution based on the 3-axial SCG/GCG recordings obtained during rest is comparable with the performance of an exercise SCG suggesting a solution that is amenable for portable at-home screening of CAD.

In our future studies, we aim to address the following limitations of the current study: (a) analysis of stenosis with different degrees of occlusion. In the current study, we investigated the possibility of identifying patients with stenosis ⩾50% in at least one coronary artery. In a future study, we will investigate the possibility of early detection of the individuals with coronary artery stenosis of a 25% occlusion rate, (b) Also, we will investigate the potential of SCG/GCG in localizing the coronary occlusion, (c) In this study, we used the fusion technique to get the final CAD risk probability for each patient. This generally indicates that our models assume that most cardiac cycles are affected by coronary artery disease. However, we need to further investigate the effect of coronary artery disease on the individual cardiac cycles to see how the disease manifests itself in each axis of vibration, (d) Within a subject, the morphology and also the frequency components of the SCG/GCG recordings varied from one cycle to another. This variation could be mainly due to the effects of breathing (Tavakolian et al., 2008). In the future study, we would like to examine the effects of coronary artery disease on the morphology of SCG/GCG cycles in the inspiration and expiration periods separately, (e) In the future study, instead of training an original model for detecting the coronary arteries, we will use a pre-trained CNN model. This technique, known as transfer learning, will improve the generalization and eliminate the need for a huge labeled data set for training a model.

## Data Availability Statement

The datasets generated for this study will not be made publicly available. Currently, this data set is in the possession of Heart Force Medical Inc. However, the authors have the intention to make the data set publicly available in the near future. The features extracted from the signals are available by request.

## Ethics Statement

The studies involving human participants were reviewed and approved by Office of Research Ethics at Simon Fraser University, Vancouver, Canada and University Hospital of Zurich. The patients/participants provided their written informed consent to participate in this study.

## Author Contributions

PD processed the data, designed and developed the models, analyzed the results, prepared the figures, and drafted the manuscript. EB provided his medical expertise in designing the models and statistical processing, analyzed the results and also revised the manuscript critically for content. KT, ZX, and AB analyzed the results and revised the manuscript critically for content. FK-K contributed to the design and development of the models and revised the paper critically for content. All authors contributed to the article and approved the submitted version.

## Conflict of Interest

PD and ZX are employed by Heart Force Medical Inc., Vancouver, Canada. EB is on the Board of Directors at Heart Force Medical, Inc. Vancouver, Canada. FK-K is the CTO of Heart Force Medical Inc., Vancouver, Canada. The remaining authors declare that the research was conducted in the absence of any commercial or financial relationships that could be construed as a potential conflict of interest.

## Publisher's Note

All claims expressed in this article are solely those of the authors and do not necessarily represent those of their affiliated organizations, or those of the publisher, the editors and the reviewers. Any product that may be evaluated in this article, or claim that may be made by its manufacturer, is not guaranteed or endorsed by the publisher.
